# Characterization and Feasibility Assessment of Recycled Paper Mill Sludges for Land Application in Relation to the Environment

**DOI:** 10.3390/ijerph120809314

**Published:** 2015-08-07

**Authors:** Rosazlin Abdullah, Che Fauziah Ishak, Wan Rasidah Kadir, Rosenani Abu Bakar

**Affiliations:** 1 Institute of Biological Sciences, Faculty of Science, University of Malaya, Kuala Lumpur 50603, Malaysia; 2 Department of Land Management, Universiti Putra Malaysia, Serdang 43400, Selangor Darul Ehsan, Malaysia; E-Mail: cfauziah@upm.edu.my (C.F.I.); rosenani@upm.edu.my (R.A.B.); 3 Soil Management Branch, Forest Research Institute Malaysia, Kepong 52109, Selangor Darul Ehsan, Malaysia; E-Mail: rashidah@frim.gov.my

**Keywords:** ^13^C-NMR spectrum, concentration, FTIR spectrum, heavy metals, land application, nutrients, soil properties

## Abstract

The disposal of industrial paper mill sludge waste is a big issue and has a great importance all over the world. A study was conducted to determine the chemical properties of recycled paper mill sludge (RPMS) and assess its possibilities for land application. RPMS samples were collected from six different paper mills in Malaysia and analyzed for physical and chemical properties, heavy metals, polycyclic aromatic hydrocarbons, ^13^C-NMR spectra and for the presence of dioxins/furans. The RPMS was dewatered, sticky with a strong odour, an average moisture of 65.08%, pH 7.09, cation exchange capacity (CEC) 14.43 cmol _(+)_ kg^–1^, N 1.45, P 0.18, K 0.12, Ca 0.82, Mg 0.73, Na 0.76 and Al, 1.38%. The polycyclic aromatic hydrocarbons (PAHs) and heavy metals levels were below the standard Class 2 limits. The dioxin and furan were in below the standard concentration of Class 1. The most prominent peak in the ^13^C-NMR spectra of RPMS was centered at 31 ppm, proving the presence of methylene (-CH_2_) groups in long aliphatic chains, with lipids and proteins. The signal at 89 ppm and highly shielded shoulder at 83 ppm were due to presence of cellulose carbon C-4, and the peak at 63 and 65 ppm was due to the cellulose carbon spectrum. The RPMS therefore contains significant amount of nutrients with safe levels of heavy metals and PAHs for environment and can be used as a fertilizer and soil amendment for land application.

## 1. Introduction

The industrial sector plays a significant role in the growth of the world economy. The paper recycling process produces a considerable amount of organic waste, which is not suitable for the production of new paper. A huge quantity of the sludge produced by paper mills with large usage of paper is considered as one of the most serious environmental problems [1]. The increasing amount of sludge and its consequent treatments are very sensitive environmental problems [2]. In Malaysia, the amount of mill solid waste produced increased from 16,200 tons per day in 2001 to 19,100 tons in 2005 or an average of 0.8 kilogram *per capita* per day. The industrial sector in Malaysia produced about 30% of solid wastes and this amount is increasing by about 4% annually [3]. The waste is also known as recycled paper mill sludge (RPMS). Recycled paper mill sludges are complex mixtures of fibrous recycled paper, inorganic solids and chemical additives used in the paper manufacturing. This sludge is the final processed waste from the pulp and paper industries which are generated from different stages of the paper making process, including the sorting, pulping, screening, cleaning, deinking, refining, color stripping and bleaching processes. Paper mill sludges are composed of organic matter (mainly cellulose fiber from wood or recycled paper) in which organic compounds are added to the paper or pulp while inorganic compounds (mainly calcium carbonate, kaolinite and talc) are also utilized [4]. 

The disposal of RPMS is an inevitable problem for these industries. It has also been reported that the main contributors to the escalating costs of waste disposal include transportation and tipping fees and the process has a negative impact on the environment, especially concerning odour and leachate. The present disposal practice is via landfill, which might not be viable in the long run as land is getting scarce with escalating cost, and this industry also faces increasingly stringent environmental regulations [5]. However, several industries take irresponsible actions to decrease the cost of disposal by illicitly dumping their waste. This situation will cause the negative pollution effect to the soil, water and air. 

The utilization of waste material in a suitable manner of application will balance the increasing demands of limited natural resources [6]. The high capital cost that is beyond the ability of small capacity mills and even then, the surplus amount of sludge is still large and has to be disposed as landfill [7]. Furthermore, direct applications on land are the preferred method of utilizing paper mill sludge which is also cost effective. 

Paper mill sludge is an active organic material that has potential benefits as a source of nutrients for crops, but potentially can pose significant environmental and public health hazards. There is also limitation for spreading paper mill sludge on agricultural land. In order to appropriately manage organic residues, it is important to thoroughly characterize their chemical and physical properties and accurately assess the impacts of these properties on soil fertility and site quality. Hence, this study was undertaken to determine the chemical properties of recycled paper mill sludges (nutrients, heavy metals content and organic contaminants) and to assess the possibilities of recycling this waste for agricultural land application in a safer manner. 

## 2. Experimental Section

### 2.1. Recycled Paper Mill Sludge Sampling

**Figure 1:**
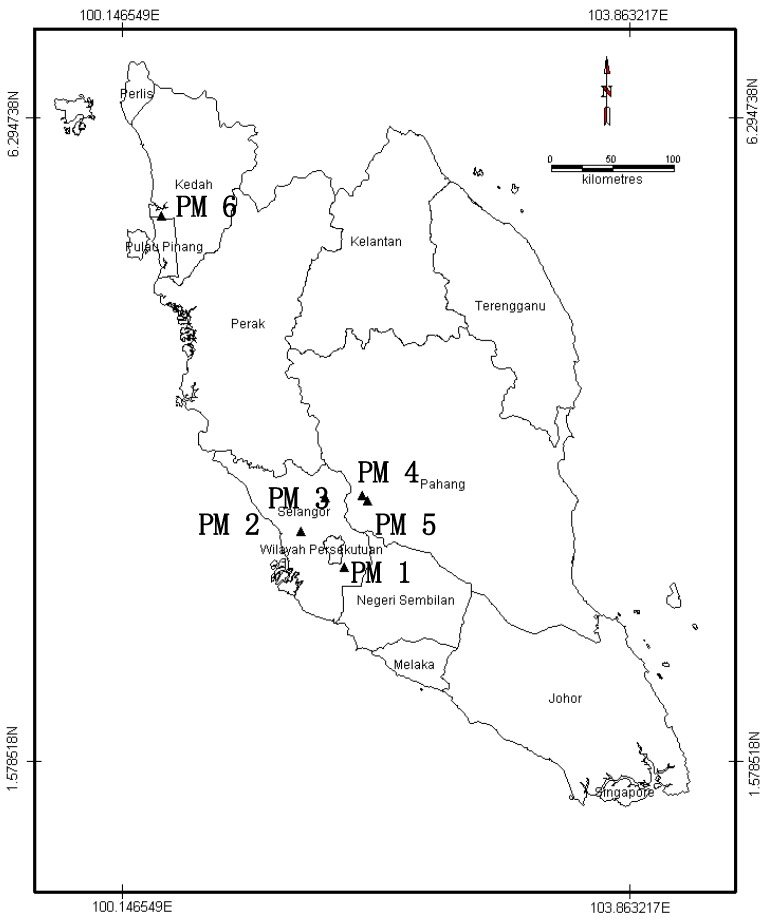
Map of the sampling locations in Peninsular Malaysia for the paper mill sludges used in this study.

Recycled paper mill sludge (RPMS) was collected from six different paper mills from the Malaysia (Figure 1) in sufficient amount for the purposes of characterization. The six samples were assigned as PM 1, PM 2, PM 3, PM 4, PM 5 and PM 6. Recycled paper mill sludge which was collected from the waste treatment plants of each mills were analyzed for physical and chemical characteristics. During the collection, the RPMS were in wet solid form. They were brought to the laboratory, air dried and ground to pass through a 2 mm sieve for the analyses of the chemical properties.

### 2.2. Physical and Chemical Characteristics of Paper Mill Sludges

The dried paper mill sludges were analyzed for moisture content, pH, organic carbon, total N and total heavy metals. The laboratory analyses were performed on the sub-samples, each in five replicates. The pH of the sludge was determined from the supernatant of the mixed sample and distilled water ratio of 1:2.5 using a Beckman Digital pH meter (Beckman Instruments Inc., CA, USA). EC was measured from a solution collected from the saturated paste using the EC meter. Total carbon was measured by the combustion technique [8] using a CR-412 carbon analyzer (LECO Corporation, St. Joseph, MI, USA). Organic matter (OM) content was calculated from loss on ignition (correction factor of 1.78) by using following formula:

OM = 1000 × (mass of oven dry soil – mass of ignited soil) / mass of oven dry soil.

Total nitrogen was determined using the modified Kjeldahl method [9]. Soil CEC was determined by the NH_4_OAc (pH 7.0) method [10]. 

### 2.3. Determination of Total Heavy Metals in Recycled Paper Mill Sludge 

The *aqua regia* method was used to extract total heavy metals in the sludge and soil [11]. *Aqua regia* is a mixture of concentrated hydrochloric acid (HCl) and nitric acid (HNO_3_) in the ratio of 3:1. Evidently, *aqua regia* is the only extractant that can release most of the components bound in the silicate matrix (residual fraction) [12]. Total metal concentrations were determined using a PE5100 atomic absorption spectrophotometer (Perkin Elmer, Wellesley, MA, USA) except for phosphorus (P) concentration in the extract which was determined using the Quikchem FIA 8000 auto-analyzer (Lachat Instruments, Loveland, CO, USA). 

### 2.4. Analytical Procedure for Polycyclic Aromatic Hydrocarbons

Determination of PAHs was carried out at the Centre of Excellence for Environmental Forensics, Faculty of Environmental Studies, Universiti Putra Malaysia (UPM). The samples were purified and fractionated according to the method for polycyclic aromatic hydrocarbons (PAHs) [13]. Glassware for the experiment were rinsed sequentially with methanol, acetone and hexane to remove any organic contaminants and kept in an oven at 60 °C. About 20 g dry weights of the sludge samples were homogenized with anhydrous sodium sulphate (Na_2_SO_4_) to remove excess water. Cellulose thimbles were filled with the samples and then transferred into the glass chamber. Round-bottom flask were filled with 300 mL dichloromethane (DCM) and then set up together with the glass chamber and heater in the extraction unit. The samples were then extracted using dichloromethane for 8 h. The extracts were transferred into a pear-shape vial from the round-bottom flask after volume of the extract was reduced to dryness by the rotary evaporator. Extracted samples were purified and fractioned into aromatic fraction through silica gel column chromatography. About 50 μL of the PAH surrogate internal injection standard mixture (10 ppm each component; anthracene-d_10_ and chrysene-d_12_) were added to the extracts. The extracts were transferred onto the top of 5% H_2_O deactivated silica gel column. The flasks with reduced sample were rinsed with 20 mL hexane: DCM (3:1 v/v). The volume of sample extracts was reduced to 2 mL using a rotary evaporator for the next step which was injection into the column chromatography. In the second step of sample introduction into the column chromatography, fully activated silica gel was used to pack the column. The PAHs fractions were eluted with 16 mL of dichloromethane/hexane (1:3, v/v). Each PAH fraction was evaporated to approximately 1 mL, transferred to a 1.5 mL amber ampule, and evaporated to dryness under a gentle stream of nitrogen and redissolved in 50 µL of isooctane containing *p*-terphenyl-d_14_ was used as an internal injection standard (IISTD) for PAHs analysis for quantification and to eliminate errors due to variation in sample injection. PAHs analyses were performed using an Agilent Technologies 5973A quadrupole mass spectrometer integrated with an Agilent 6890 gas chromatograph (Agilent Technologies, Santa Clara, CA, USA). A 30 m fused silica capillary column of 0.25 mm i.d. and 0.25 µm film thickness using helium as carrier gas was carried in the analysis. GC-MS operating conditions were set at 70 eV ionization potential with the source at 200 °C and electron multiplier voltage at ~2000 eV. The injection port was maintained at 310 °C and the sample was injected with splitless mode followed by purging for 1 min after the injection. Column temperature was held at 70 °C for 2 min, then programmed at 30 °C/min to 150 °C, and then 4 °C/min to 310 °C and held for 10 min. A selected ion monitoring method was employed after a delay of 4 min. PAHs were monitored at *m/z* = 178 (phenanthrene anthracene), *m/z* = 192 (3-methylphenanthrene, 2-methylphenanthrene, 2-methyl-anthracene, 9-methylphenanthrene, 1-methylphenanthrene), *m/z* = 202 (fluoranthene, pyrene), *m/z* = 216 (1-methylpyrene), *m/z* = 228 (chrysene, benzo[a]anthracene), *m/z* = 252 (benzo[k]fluoranthene, benzo[e]acephenanthrylene, benzo[e]pyrene, benzo[a]pyrene), *m/z* = 278 (dibenzo[a,h]anthracene). 

### 2.5. Analytical Procedure for Dioxin/Furan

Determination of Dioxin/Furan was carried out by the Doping Control Centre (DCC), Universiti Sains Malaysia (USM), Pulau Pinang, Malaysia. MA modified method was developed according to USEPA 8290 and 1613. The dioxin/furan analyses were carried out using gas chromatography-high resolution mass spectrometry (GC-HRMS). Only one paper mill sample was used to determine the dioxin/furan levels, which was then used to represent the dioxin/furan content for sludge produced in Malaysia. Paper Mill 2 (PM 2) was selected because this sludge was also used for further studies (laboratory, glasshouse and field conditions).

## 3. Results and Discussion

### 3.1. Physico-Chemical Characteristics of Paper Mill Sludges

**Figure 2:**
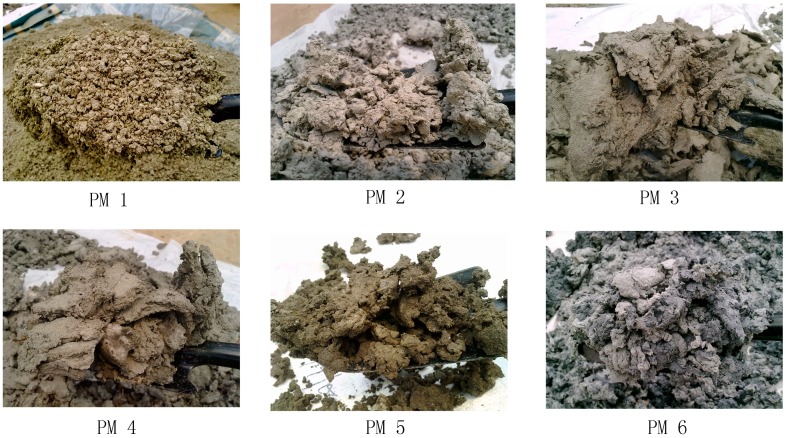
Physical appearances of different paper mill (PM) sludges from Peninsular Malaysia.

The paper mill sludges produced by the paper industries in Malaysia were wet, sticky and had a strong odour (Figure 2). Almost all mill sludges which was collected from the various mills has similar characteristics, except for the contents of nutrients and heavy metals which varied from each other. The moisture content in the sludge ranged from 45.78%–78.32% with an average of 65.08%. Pulp and paper mill sludges were generally dewatered mechanically to increase the solids content, reduce the volume and weight and improve their handling properties (Table 1). The composting of paper mill is recognized as the most adequate pre-treatment in order to obtain a material which may respond more efficiently with reduced odour and can help sanitize the material [14].

The mean pH value of the paper mill sludges was 7.09 (Table 1). The alkalinity of paper mill sludges typically arises from causticizing materialsused in the pulping process and/or CaCO_3_ used in the paper finishing process. Most of the agricultural soils in Malaysia are acidic in nature with low soil pH. Hence, in the sludge contains CaCO_3_ that could be helpful for the soil improvement. In addition, paper sludge is suitable for land application because CaCO_3_ can help neutralize soil acidity due to the cellulosic fiber content that can hold moisture in the soil system. The electrical conductivity values of the six paper mill sludges ranged from 0.51–3.08 mS·cm^−1^. An EC level of < 2 mS·cm^−1^ in soil and irrigation water is generally considered a safe level for plants. However, most of the plants can tolerate soils with an EC of 3–4 mS·cm^−1^. 

The CEC values of paper mill sludges ranged from 3.33–28.07 cmol _(+)_ kg^–1^. Average CEC content is 14.43 cmol _(+)_ kg^–1^ (Table 1). Reported CEC values of paper mill sludges from earlier studies varied widely from 5.3 to 297 cmol _(+)_ kg^–1^ [15,16,17,18,19]. Differences in CEC may reflect differences in sludge composition, organic matter fraction and/or clay content [20].

The N content of the paper mill sludges ranged from 0.31% to 4.05%. Average N content was 1.45% (Table 1). In this study, the P content of paper mill sludges ranged from 0.02 to 0.78% with an average P content is 0.18%. Nitrogen and P, which are essential for microbial metabolism, are typically added to wastewater during the secondary treatment process which increases the N and P concentrations of the paper mill sludges [21] with N contents ranging from 0.6% to 8.8% with a median value of 2.3% [22]. Industrial wastewaters from operations like detergent manufacturing and metal coating processes may present P levels higher than 10 mg·L^−1^ [23]. The carbon content of the paper mill sludges ranged from 18.92% to 33.67% with an average value of 25.61%. The C:N ratios of the paper mill sludges were calculated to be 8.31–75.48. The National Council for Air and Stream Improvement [24] reported that the C:N ratios for paper mill sludge can range from 6 to 115:1. The organic matter content of paper mill sludges ranged from 32.54% to 57.91% with an average value of 44.04%. The high organic matter content is an extra benefit for the soils that will improve soil fertility and enhance the soils’ physical properties. 

**Table 1:** Physico-chemical characteristics of paper mill sludges from different paper mill industry sites.

No.	Parameters	* PM 1	PM 2	PM 3	PM 4	PM 5	PM 6	Min.	Max.	Mean
1.	Moisture (%)	69.32	78.32	59.08	68.68	69.34	45.78	45.78	78.32	65.08
2.	pH	6.45	7.84	7.32	7.54	6.33	7.08	6.33	7.84	7.09
3.	EC, mS/cm	1.12	3.08	0.51	0.61	1.97	0.56	0.51	3.08	1.30
4.	Nitrogen, %	1.29	4.05	1.51	0.31	1.32	0.49	0.31	4.05	1.49
5.	Carbon, %	24.39	33.67	18.92	23.40	31.36	21.92	18.92	33.67	25.61
6.	C/N ratio	18.91	8.31	12.52	75.48	23.75	44.73	8.31	75.48	30.61
7.	Organic matter, %	41.95	57.91	32.54	40.24	53.93	37.70	32.54	57.91	44.04
8.	CEC, cmol_(+)_ kg^−1^	25.00	28.07	4.39	3.33	18.66	7.11	3.33	28.07	14.43
9.	Phosphorous, %	0.07	0.78	0.02	0.12	0.09	0.02	0.02	0.78	0.18
10.	Potassium, %	0.13	0.42	0.02	0.05	0.07	0.06	0.02	0.42	0.12
11.	Calcium, %	0.54	0.53	0.54	1.28	0.74	0.36	0.36	1.28	0.66
12.	Magnesium, %	0.73	0.45	0.42	0.41	1.06	0.57	0.41	1.06	0.61
13.	Sodium, %	0.69	0.78	0.58	0.64	0.45	0.88	0.64	0.88	0.67
14.	Aluminum, %	1.65	2.76	1.09	1.39	1.73	1.45	1.09	2.76	1.68

* PM = Paper mill.

**Table 2:** Heavy metals content of recycled paper mill sludges of different paper mill sites.

No.	Parameters	* PM 1	PM 2	PM 3	PM 4	PM 5	PM 6	Min.	Max.	Mean				Zarcinas, (2004) **

											^#^ Class 1	^#^ Class 2	^#^ Class 3	
1.	Cadmium, mg·kg^−1^	2.01	4.09	2.07	2.47	1.3	1.38	1.3	4.09	2.34	3	20	20	0.3
2.	Chromium, mg·kg^−1^	18.92	37.01	7.44	17.36	26.56	12.92	7.44	37.01	20.58	100	1060	1060	60
3.	Copper, mg·kg^−1^	199	102	119	102	156	83	83	199	130.38	100	757	757	50
4.	Manganese, mg·kg^−1^	241	329	85	343	102	103	85	329	203.88	-	-	-	-
5.	Lead, mg·kg^−1^	81	328	43	61	73	55	43	328	126.5	150	500	500	65
6.	Zinc, mg·kg^−1^	358	287	257	277	365	351	257	365	314.63	500	1850	1850	95
7.	Nickel, mg·kg^−1^	41.6	29.11	13.20	10.14	15.96	10.78	10.14	4.16	21.56	62	180	180	45
8.	Iron, %	0.41	0.38	0.19	0.42	0.37	0.37	0.19	0.42	0.34	-	-	-	-

* PM = Paper mill, **^#^** Classifications were followed for land spreading of pulp and paper mill sludge by the British Columbia Pulp and paper Association [25], ** Investigation Level for Malaysian soils which the level is taken at the 95th percentile of the heavy metals data for agricultural soils [26].

The K content of the paper mill sludges ranged from 0.02% to 0.42% with an average K content of 0.12% (Table 1) that shows its lower quantity. Generally, pulp and paper mill sludges do not provide significant quantities of K [27].

The Ca and Mg contents ranged from 0.36%–1.28% and 0.41%–1.06%, respectively (Table 1). Pulp and paper mill sludges, often contain significant quantities of Ca, and sometimes Mg, in the form of carbonates and hydroxides, and have been proven to effectively neutralize soil acidity. In projects where pulp and paper mill sludges were used to reclaim mine tailings, the liming effect of the pulp and paper mill sludges have proven to be one of the primary benefits to soil fertility [28].

In the paper mill sludge, Na content ranged from 0.64% to 0.88%. Meanwhile, the Al content ranged from 1.09%–1.68% (Table 1). Sodium and Al can occur in pulp and paper mill sludges at levels higher than would typically be found in non-amended soils. However, some studies to monitor these higher contents of Na and Al have been carried out for land application with these residuals [29]. Sodium is used in the pulping process as sodium hydroxide and Al is associated with the use of clays in the paper making process and the use of Al salts (e.g., aluminum sulfate) in the wastewater treatment process [30].

### 3.2. Heavy Metals Concentrations of Recycled Paper Mill Sludges

The heavy metals concentrations in the paper mill sludge is one of the major issues as it needs to be proven that either heavy metals are above the critical limits or not, which may increase the concentration in the soil. After the analysis it was found that the concentration of heavy metals in the paper mill sludge varied among different paper mills (Table 2). In this study, guidelines for land spreading of pulp and paper mill sludge which were followed by the British Columbia Pulp and paper Association, Lands and Parks [25] were used. From the regulation, only paper mill sludges of Class 1 and Class 2 can be allowed for application on agricultural lands.

Total concentrations of heavy metals in the six paper mills, with the exception of Cr, were above the Investigation Level for Malaysian soils [26], which is the level taken at the 95th percentile of the heavy metals data for agricultural soils of Malaysia. Camberato *et al.* [21] surveyed several organic residues, including pulp and paper mill sludges and biosolids, and reported that the levels of the regulated heavy metals in pulp and paper mill sludges were lower than those found in biosolids, but slightly higher than the “background” levels of these metals in soils.

The Cu content of paper mill sludges ranged from 83 to 199 mg·kg^−1^ with an average of 130.38 mg·kg^−1^ which is above the Class 1 limit of 100 mg·kg^−1^. Beauchamp *et al.* [31] reported that concentration of Cu in paper mill sludge varied between 84 and 118 mg·kg^−1^. Copper was determined in different chemical additives in the paper making process, and the analyses of the inks (cyan ink) showed a concentration of 10,685 µg·g^−1^ of Cu. Therefore, the source of Cu content in the paper mill sludge is likely due to the presence of cyan ink. 

The lead (Pb) content of paper mill sludges ranged from 43 to 328 mg·kg^−1^ with an average of 126.5 mg·kg^−1^ which is also above the Class 1 limit of 150 mg·kg^−1^. In this study, the Cd content of paper mill sludges ranged from 1.3 to 4.09 mg·kg^−1^ (which is above the Class 1 limit of 3 mg·kg^−1^) with an average Cd content of 2.34%. Concentrations of Cr, Zn and Ni in the paper mill sludge of the six mills were well below the standard concentration of Class 1. 

The average total concentration of Mn and Fe in six paper mill sludges analyzed were 203 mg·kg^−1^ and 0.34%, respectively. No standard was available for concentrations of Mn and Fe. However, these metals are valuable micronutrients, therefore, the agronomic application rates were taken into consideration. According to Murray [32], the presence of Fe is more likely linked to the addition of kaolin, the clay used to coat paper which contains 1% Fe_2_O_3_. Furthermore, Fe content in paper mill sludge is negligible compared to the level found in soil, that is lesser than 50,000 µg·g^−1^ [33].

Heavy metal levels in pulp and paper mill sludges are generally low enough that the application rates to soil will not be limited by the heavy metals content [34,35]. According to Camberato *et al.* [21], paper mill sludges generally have lesser metal concentrations than municipal waste biosolids and well within regulatory limits. 

Similar results were reported by Li *et al.* [36] and Lu *et al.* [37] in that the metals of prepared paper mill sludges the major toxic metal was Ba, followed by Cu, Zn, Pb and Cr, which were lower than the thresholds prescribed in GB 5085.3-2007. The use of these types of sludges is not harmful for the soil and plants due to have less concentrations of the heavy metals and could be useful as soil amendments. 

### 3.3. Organic Contaminants of Polycyclic Aromatic Hydrocarbons

Seventeen polycyclic aromatic hydrocarbons (PAHs) were analyzed in this study (Table 3). The PAHs have received special attention since they have long been recognized as hazardous environmental chemicals. The total concentrations of the 17 PAHs found in the six paper mill sludges were within the range of 218.04 to 3646.67 ng·g^−1^. The highest concentration of total PAHs were recorded for sludge from Paper Mill 3 and the lowest was recorded for Paper Mill 2. The total PAHs in raw paper mill sludge was less than 6 ng·g^−1^ (the value recommended by the draft directive of the European Union for the land disposal of sludges) [31]. Seventeen PAHs were detected at concentrations below the standard of Class 2 followed by the British Columbia Pulp and paper Association, Lands and Parks [25]. In addition, the paper mill sludges showed lower PAHs concentration than some Canadian sewage sludges (traced to 100 ng·g^−1^) that were used in agriculture [38]. 

### 3.4. Dioxins and Furans

The presence of trace organic components in pulp and paper solid residues either as contaminants or by-products of the particular process has been an area of concern for regulatory agencies and the general public. A primary concern in the land application of pulp and paper mill residues is due to the presence of trace amounts of chlorinated dioxins and furans. The concentration of dioxins and furans in the Paper Mill 2 sludge was 4.32 pg·g^−1^, which is well below the Class 1 standard (10 pg·g^−1^). The low dioxin content makes paper mill sludge a potential organic material for land application in Malaysia. The presence of total dioxin equivalents varied from 1 to 48 ng·TEQ·kg^−1^ in Ontario (39) and from 2 to 14 ng·TEQ·kg^−1^ in Quebec. Meanwhile, the Maine Department of Environmental Protection (USA) has stated that sludges containing 27 pg·g^−1^ or lower are approved for agricultura1 applications, while levels of 28–250 pg·g^−1^ are restricted to non-agricultural uses [39]. The RPMS has good potential to manage industrial wastes as a resource through wastes recovery that will create alternative resources for landfilling [40] and will minimize the negative impact of waste on the environment and human health.

**Table 3:** Concentrations (ng·g^−1^, dry weight) of individual polycyclic aromatic hydrocarbons compounds in recycled paper mill sludges of different Malaysian paper mills.

No.	* PAHs	^#^ PM 1	PM 2	PM 3	PM 4	PM 5	PM 6	Class 1	Class 2	Class 3
								(µg·g^−1^)		
1.	Phenanthrene	113.76	39.10	532.72	n.d	77.33	n.d	0.1	50	50
2.	Anthracene	91.09	61.92	703.13	n.d	159.24	n.d	0.1	10	10
3.	3-Methylphenanthrene	158.79	18.55	265.65	256.47	244.21	211.84	0.1	10	10
4.	2-Methylphenanthrene	152.61	16.24	279.95	351.67	301.30	80.21	0.1	10	10
5.	2-Methylanthracene	26.80	n.d	53.40	n.d	59.55	n.d	0.1	10	10
6.	9-Methylphenanthrene	15.43	n.d	251.34	192.97	243.00	n.d	0.1	10	10
7.	1-Methylphenanthrene	101.79	n.d	193.56	137.95	202.51	n.d	0.1	10	10
8.	Fluoranthene	168.95	13.98	86.54	4.32	84.33	40.25	0.1	10	10
9.	Pyrene	278.38	14.68	165.03	23.33	146.77	58.27	0.1	100	100
10.	1-Methylpyrene	478.92	37.00	337.59	58.13	273.87	360.86	0.1	10	10
11.	Chrysene	56.79	n.d	23.22	7.99	19.86	18.42	0.1	10	10
12.	Benzo(a)antharene	138.98	16.57	38.32	11.65	46.79	46.87	0.1	10	10
13.	Benzo(k)fluoranthene	0.79	n.d	0.68	0.87	1.41	0.88	0.1	10	10
14.	Benzo(e)acephenanthrylene	1.05	n.d	0.64	0.67	1.63	1.01	0.1	10	10
15.	Benzo(e)pyrene	0.79	n.d	0.99	1.51	1.06	1.30	0.1	10	10
16.	Benzo(a)pyrene	2.62	n.d	0.95	0.68	2.62	3.15	0.1	10	10
17.	Dibenzo(a,h)antharacene	5.92	n.d	19.71	2.98	23.60	23.27	0.1	10	10
	Total PAHs	1793.45	218.04	3646.67	1051.20	1889.10	846.33			

* PAHs = polycyclic aromatic hydrocarbons (PAHs), **^#^** PM = paper mill, n.d- the value of the PAHs were not detected in the analysis. Classifications were followed for land spreading of pulp and paper mill sludge by the British Columbia Pulp and paper Association [25].

### 3.5. ^13^C-NMR Spectra of Paper Mill Sludge

**Figure 3:**
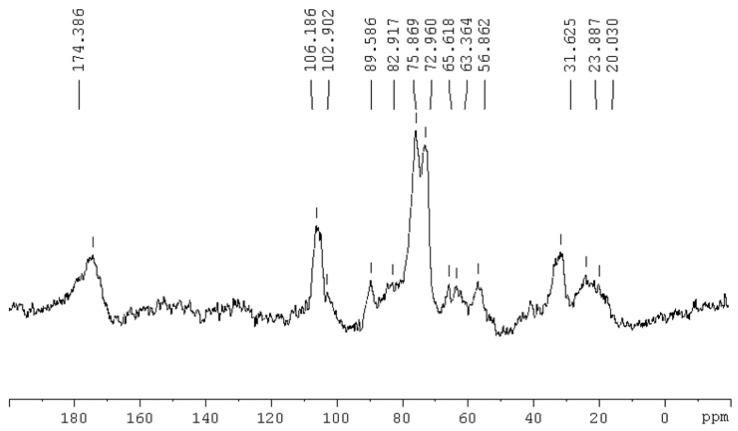
The ^13^C-NMR spectra of raw RPMS.

Among the various paper mills it was found that PM 2 was the better sludge compared to the other as it has high in moisture content, pH, EC, C, OM, CEC, lower in heavy metals, PAHs, and dioxins and furans. With these all properties it can be utilized for the improvement of acidic soils (most of the agricultural soils in Malaysia) and can be selected for further use in laboratory, glasshouse and field studies. Hence, only PM 2 was chosen to record the ^13^C-NMR spectra due to its better suitability for land application (Figure 3). The spectrum of raw RPMS showed the presence of peaks at 20, 23, 31, 56, 63, 65, 72, 75, 83, 89, 102, 106 and 174 ppm. Jackson and Line [41] reported a similar ^13^C-NMR spectrum for paper mill sludge in their study. The peaks at 20, 23 and 31 ppm are attributable to non-substituted alkyl carbons. The most prominent peak was centered at 31 ppm, which suggests the presence of methylene (-CH_2_) groups in long aliphatic chains, with a possible contribution from lipids and proteins too. Since no phenolic signals were present in the spectrum (145–160), the RPMS could be assumed to contain little or no lignin. The only signal that may indicate the presence of lignin was at 56 ppm which is usually assigned to methoxyl groups (OCH_3_) associated to lignin and lignin-like products [41]. 

The presence of N alkyl C may also contributed to this resonance region. The large doublet between 72 and 75 ppm was due to the C-2, C-3 and C-5 carbons of cellulose and the peak at 63 and 65 ppm was due to the cellulose C-6 carbon. The signal at 89 ppm and highly shielded shoulder at 83 ppm were due to presence of cellulose carbon C-4. Usually the peaks at 72 and 106 ppm are thought to correspond to resonance of carbons in polysaccharides rings and to anomeric carbons in polysaccharides [42]. Furthermore, Schmidt *et al.* [43] observed the signal at 72 and 106 ppm together with a shoulder around 65 to 90 ppm, most likely due to polysaccharides. The peak at 103 ppm was due to hemicellulose. The high resonance at 174 ppm may arise from phenolate, carboxylic and amide groups [44]. The ^13^C NMR obtained in this study suggested that the RPMS consisted of chemically isolated cellulose and contains little lignin plus hemicellulose-like material. The high cellulose content in the paper mill sludge may be accounted for in part by mechanical and chemical degradation occurring during paper manufacture and bleaching. The lack and comparatively low level of lignin was due to the bleaching process. The mechanical degradation of polysaccharides during paper manufacturing may thus be responsible for the production of paper mill sludge with a high cellulose and low lignin content. 

### 3.6. FTIR Spectra of Paper Mill Sludge

**Figure 4:**
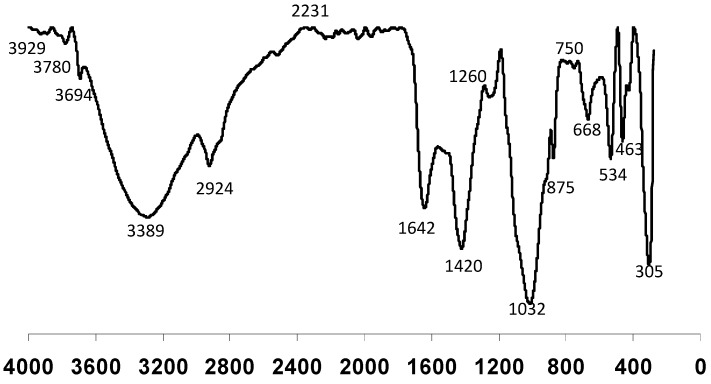
The FTIR spectrum of paper mill sludge.

The FTIR spectrum complemented the structural information obtained in the ^13^C-NMR spectrum. Infrared spectroscopy is a technique used to identify various functional groups in unknown substances via the identification of bond vibrations at designated wavelengths. The FTIR spectrum for the raw RPMS of Paper Mill 2 (PM 2) (Figure 4) showed a broad band around 3389 cm^−1^ corresponding to OH stretching of phenolic OH groups. The band attributed to the hydroxyl group and water at 3700–4000 cm^−1^ was also detected in the raw and RPMS compost. The moisture of raw and RPMS compost was found to be higher than the mineral soil. However, the intensity was too weak and considered insignificant. A distinct peak at 2924 cm^−1^ corresponding to C-H stretching of the CH_2_ groups, indicates the presence of various amino acids. This band may also be the characteristic for the presence of aliphatic methylene groups in these compounds. The weak C≡C stretching band of alkyne molecules normally occurs in the region of 2231 cm^−1^. The C=O stretching at peak 1642 cm^−1^ was protein of amide I’s origin. Giovanela *et al.* [45] suggested that nitrogen bands in the IR spectrum are mostly due to the presence of amide groups. Aliphatic C-H bending was observed with the peak at 1420 cm^−1^. The band at 1260 cm^−1^ can be assigned to the C-O stretch of carboxylic acids and to the C-N stretch of amides (amide III). According to the findings of Matias *et al.* [46], the peaks detected around 1260 cm^−1^ were typical of lignin. A very strong peak detected near 1014 cm^−1^ was assigned to C-O stretching of polysaccharide-like substances. A sharp band at 875 cm^−1^ was assigned to the C-O out of plane bend of carbonates [47]. The stretching vibration assigned to the C-S linkage occurred in the region at 700–600 cm^−1^. Meanwhile, brominated compounds appeared in the 600–500 cm^−1^ infrared band region [48]. 

## 4. Conclusions

The study demonstrated that recycled paper mill sludge has potential and can be used as a fertilizer applied for soil amendment. The application of recycled paper mill sludge to tropical acidic soils can provide substantial benefits such as neutralization of soil acidity, increased organic matter and other essential nutrients. Composting may be a viable option for recycled paper mill sludge as it can enhance its quality, since the physical properties of paper mill sludge are practical. However, the uptake of heavy metals by crops and the fate of these heavy metals in soils should be monitored to avoid the potential for soil and water pollution.
